# Head-to-Head Comparison of 5 Anti-SARS-CoV-2 Assays Performance in One Hundred COVID-19 Vaccinees, over an 8-Month Course

**DOI:** 10.3390/diagnostics12061426

**Published:** 2022-06-09

**Authors:** Jakub Swadźba, Tomasz Anyszek, Andrzej Panek, Agnieszka Chojęta, Kinga Wyrzykowska, Emilia Martin

**Affiliations:** 1 Medical Department Diagnostyka S.A., 31-864 Krakow, Poland; jakub.swadzba@diag.pl (J.S.); tomasz.anyszek@diag.pl (T.A.); andrzej.panek@diag.pl (A.P.); agnieszka.chojeta@diag.pl (A.C.); kinga.wyrzykowska@diag.pl (K.W.); 2 Department of Laboratory Medicine, Faculty of Medicine and Health Sciences, Andrzej Frycz Modrzewski Krakow University, 30-705 Krakow, Poland

**Keywords:** COVID-19, SARS-CoV-2, vaccination, assay comparison, humoral immunity, immunoassay, antibodies

## Abstract

The immunoassays used to measure anti-spike SARS-CoV-2 antibodies are widely available on the market. However, their performance in COVID-19 vaccinees is not yet adequately assessed. Our study provides a head-to-head comparison of five methods: Abbott’s S1-RBD IgG, Roche’s S1-RBD total antibody, Euroimmun’s S1 IgG, and DiaSorin’s TrimericS IgG and S1/S2 IgG assays. Testing was performed in one hundred vaccinated subjects, at eight timepoints over eight months after vaccination. The results differed substantially between methods; however, they correlated strongly and demonstrated the individuals’ responses to both doses of vaccination and the waning of humoral immunity after eight months. Importantly, we encountered a high percentage of results above the assay-specific upper quantitation limit (UQL) for undiluted samples. This was the most pronounced for the Roche’s and Euroimmun’s assays. The Abbott’s assay showed the lowest percentage of results above the UQL. We also attempted to find a common way to establish antibody concentrations that might be classified as high. However, this resulted in between 10% and 100% of such results for different methods on day 240′. This highlights the need for an assay-specific approach for adjusting the cut-offs that may indicate COVID-19 immunity.

## 1. Introduction

The role of serological testing evolved over the course of the coronavirus disease 2019 (COVID-19) pandemic. Severe acute respiratory syndrome coronavirus 2 specific antibody tests (anti-SARS-CoV-2) were initially used as an auxiliary means of disease diagnosis [1,2,3]. Once direct pathogen recognition using either molecular or antigen testing became more available, the utility of the serologic assays was shifted to the field of epidemiological studies [4]. The advent of anti-COVID-19 vaccinations re-ignited interest in serological testing. Clinical trials utilized antibody testing in vaccine immunogenicity studies. Then, after the general implementation of vaccinations, anti-SARS-CoV-2 assays started to be used to assess individual humoral responses to inoculation [5]. This is particularly important in certain groups of patients, such as the elderly or immunosuppressed [6,7]. 

The multidimensional utility of anti-SARS-CoV-2 tests enforced rapid evolution in their characteristics. Simple lateral flow assays, generating qualitative results, were shortly superseded by laboratory-based automated methods [8,9]. Further improvements focused on antibody specificity [10,11,12]. This is particularly important, considering that only the assays based on SARS-CoV-2 spike (S) protein are able to detect the humoral response to the vaccination [13]. Nucleocapsid-based (N, NCP) kits are not widely used in the vaccination era. Their sole utility may be to discern natural infection from inoculation in vaccinated subjects [14,15]. 

The aim of this study is to compare commercialized immunoassays’ performances in the monitoring of the humoral response to mRNA anti-COVID-19 vaccination. This narrowed the choice of methods included to the spike protein-based immunoassays. The main differences between the methods are determined by the exact antigenic specificity (trimeric spike, its subunits, or solely RBD-S1 domain) and immunoglobulin class of a given assay. The introduction of the first WHO International Standard (IS) for anti-SARS-CoV-2 immunoglobulin binding activity (NIBSC 20-136) was the first step towards the harmonization of results obtained using different methods, allowing for the expression of results in the standardized unit—Binding Antibody Unit/mL (BAU/mL). However, even methods validated against the standard have different cut-off levels and quantitation ranges expressed in BAU/mL. It should be noted that the WHO standard can only be used to assist the comparison of assays detecting the same class of immunoglobulins with the same specificity [16]. 

The data are lacking for the tests’ performances in vaccinated subjects. Most of the manufacturers’ validation studies, as well as the clinical evaluations, e.g., [17,18,19], were based on samples from COVID-19 patients; limited data are available from COVID-19 vaccinated subjects. If the assay’s clinical performance in this context is provided, then it focuses on sensitivity, i.e., the ability of a given test to detect the humoral response shortly after vaccination. The sole seropositivity does not directly translate to immunity [20]; efforts are being made to estimate the level of antibodies which may protect against the symptomatic disease. This crucial question is difficult to answer, as the immunoassays measuring the antibodies differ.

Our study provides some insights into the real-life performance of five different commercially available anti-SARS-CoV-2 antibody assays over a long course, eight months after the first dose of COVID-19 vaccine administration. We intend to demonstrate strengths and limitations of the analyzed immunoassays which influence their utility for monitoring the humoral response to COVID-19 vaccination. 

## 2. Materials and Methods

### 2.1. Study Design

The study participants (n = 100) were recruited from patients undergoing vaccination against COVID-19. Other than an age below 18 years, there were no exclusion criteria for participation in this study. All subjects received the Pfizer-BioNTech Comirnaty vaccine, with two doses administered 21 days apart. The cohort included 86 females and 14 males with a mean age of 45 years (23–74). There was no statistically significant difference in age between the sexes. Fifteen participants were classified as COVID-19 convalescents; eight were confirmed by positive swab between 2 and 10 months prior to day 0′, and the rest by seropositivity on day 0′ (before vaccination).

Venous blood samples were obtained via venipuncture into Greiner tubes (VACUETTE^®^ TUBE CAT Serum Clot Activator). Serum was separated by centrifugation within 1 h after sample collection. Phlebotomy was performed on day 0′, 10′, 20′, 30′, 60′, 90′, 120′, and 240′ after the first dose of the vaccine. Hence, the study covers a period of 8 months since the first dose administration, or 7 months after the complete vaccination. All of the subjects provided informed consent for participation in the study and filled out a questionnaire about their history of COVID-19. Ethical approval of the study was obtained from the Bioethics Committee of Andrzej Frycz Modrzewski Krakow University, Krakow, Poland.

### 2.2. Laboratory Testing

The DiaSorin LIAISON^®^ SARS-CoV-2 TrimericS IgG immunochemiluminescent assay was performed on fresh blood sera on the day of blood collection using the LIAISON^®^ XL analyzer (DiaSorin S.p.A, Saluggia, Italy). The remaining sera were aliquoted and frozen (−20 °C) for testing using the other investigated methods. All assays were run strictly adhering to the manufacturers’ instructions.

The Abbott SARS-CoV-2 IgG II Quant chemiluminescent microparticle immunoassay (CMIA) was performed on the Alinity i analyzer (Abbott, Sligo, Ireland); the Roche Elecsys^®^ Anti-SARS-CoV-2 S electrochemiluminescence immunoassay (ECLIA) was performed using the Cobas 8000 analyzer (Roche Diagnostics GmbH, Mannheim, Germany); the Euroimmun anti-SARS-CoV-2 QuantiVac enzyme-linked immunosorbent assay (ELISA) IgG was performed using a fully automated ELISA system EuroLabWorkstation 45 (Euroimmun, Luebeck, Germany); and the DiaSorin LIAISON^®^ SARS-CoV-2 S1/S2 IgG immunochemiluminescent assay was performed using the LIAISON^®^ XL analyzer (DiaSorin S.p.A, Saluggia, Italy). With the exception of the LIAISON^®^ SARS-CoV-2 S1/S2 IgG assay, all tests included in this study were validated against the first WHO International Standard (IS) for anti-SARS-CoV-2 immunoglobulin; their results may be expressed in Binding Antibody Units (BAU/mL). The LIAISON^®^ SARS-CoV-2 S1/S2 IgG results presented in this study are expressed in Arbitrary Units (AU/mL). 

The assays included in this study were correlated by their manufacturers using the various neutralization tests to find the level of antibodies indicating a high neutralization capability. Roche validated the S1-RBD total antibody assay results in surrogate neutralization assay GenScript^®^ cPass™ and found a correlation with high neutralization capability for antibody concentrations higher than 15.43 BAU/mL. DiaSorin showed that the TrimericS assay result of 520 BAU/mL correlated with the microneutralization titer of 1:80, and the S1/S2 IgG assay result of 80 AU/mL indicated a titer of 1:160 in a plaque reduction neutralization test (PRNT). Logistic regression analyses performed by Abbott revealed that the assay’s result of 136 BAU/mL corresponds to the PRNT dilution of 1:80 and that a result of 490 BAU/mL corresponds to the PRNT titer of 1:160. Euroimmun’s assay’s results were correlated to the PRNT results, but only qualitatively (no association between Euroimmun’s assay results and neutralizing titers was provided). As a result of the described differences in the manufacturers’ approaches to finding the threshold for high antibody titers which may have neutralization capabilities in vivo, we proposed the adjusted cut-offs for such results, calculated by multiplication of respective positivity thresholds by a constant factor. We determined the ratios of UQL/positivity cut-off for all the methods included in this study; the lowest ratio, 10.9 (obtained for the Euroimmun’s assay), was used as a constant multiplication factor.

In this study the samples for which the results exceeded the upper quantitation limit (UQL) were not diluted; their results were considered to be equal to the respective upper quantitation limit for each test. A summary of the important assays’ characteristics, including a calculated, adjusted cut-off value for a high result, is presented in the Table 1.

### 2.3. Statistical Analyses

Student’s *t*-test was used to verify the statistical significance of the difference in age between the sexes. Correlation between test results was assessed using Spearman’s coefficient of correlation. The significance level was set to 0.05.

The statistical analyses were performed using STATISTICA software ver. 13 (TIBCO Software Inc., Palo Alto, CA, USA) and R version 4.1.2 [21]. 

## 3. Results

**Figure 1:**
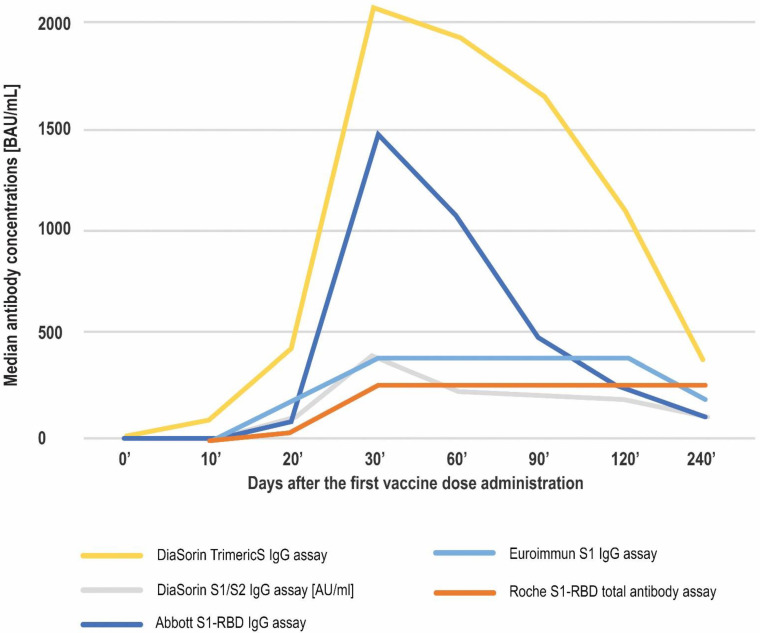
The course of anti-spike SARS-CoV-2 antibodies production over 8 months after the first vaccine dose administration. Apart from DiaSorin’s S1/S2 assay, which is expressed in AU/mL, the median concentrations are given in BAU/mL.

The results obtained using all of the studied methods proved the immunogenicity of the vaccine. An increase in antibody concentrations was observed shortly after the first vaccine dose administration, but the most pronounced rise in antibody concentrations was noted between days 20′ and 30′. Over the course of the study, the highest median concentrations were observed on day 30′, approximately 10 days after the second dose administration. The samples in the study were not diluted and in four of the methods investigated (Abbott’s test being the exception) the median antibody concentrations at this timepoint exceeded the respective upper limit of quantitation (UQL). Following day 30′, the median values started to decrease. However, the medians noted for the Roche assay remained over the UQL until day 240′, and for the Euroimmun assay until day 120′ (Figure 1, Table 2). 

**Figure 2:**
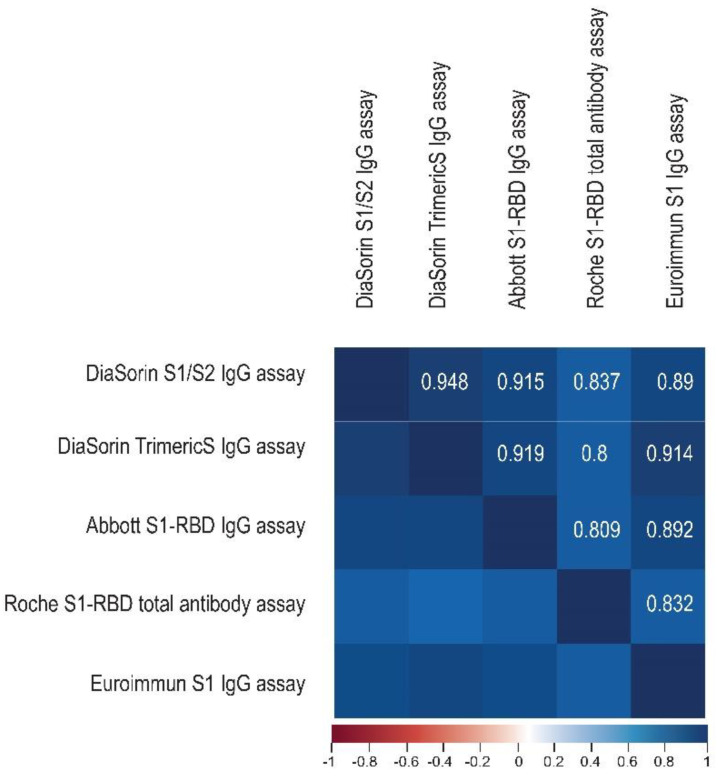
The correlation matrix heatmap shows the values of the Spearman correlation coefficient for the studied immunoassays, with the positive values (r > 0) in blue. The darker the shade of blue, the stronger the positive correlation.

We assessed the correlation between the methods using Spearman’s coefficient of correlation. The overall correlation between the tests was strong to very strong. The highest overall correlations were noted for the two DiaSorin assays (0.948), and these pairs: DiaSorin TrimericS–Abbott (0.919), DiaSorin S1/S2–Abbott (0.915), and DiaSorin TrimericS–Euroimmun (0.914). All correlations between the Roche test and the other assays were lower than r = 0.9 (Figure 2).

**Figure 3:**
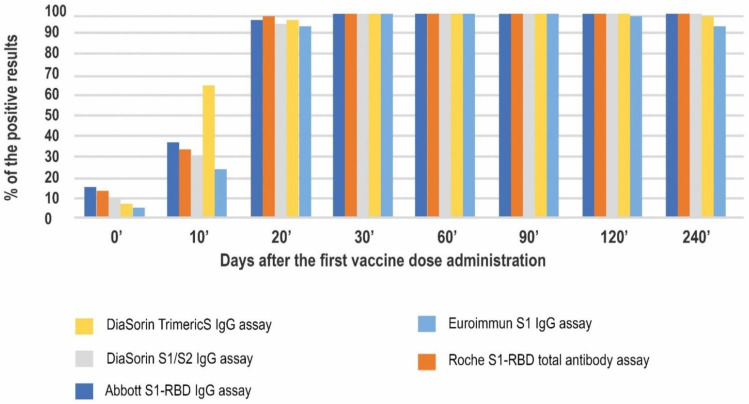
Seropositivity rates in 5 studied immunoassays at 8 timepoints of the study.

The above results provide a similar pattern of the humoral response obtained by the investigated methods and their general agreement. Further, we decided to scrutinize the differences between the tests. Firstly, we compared the percentage of positive results at the consecutive timepoints (Figure 3). 

All of the positive results observed on day 0′ were obtained from the COVID-19 convalescents’ samples. A total of 100% and 93% of the convalescents tested positive in the Abbott’s and Roche’s tests, respectively. The lowest percentage of positive results in the convalescent group on day 0′ was observed for Euroimmun (40%). Of interest, not one of the convalescents consistently tested positive in all of the methods used. On day 10′, positive results started to be noted in naïve vaccinees as well. At this timepoint, the DiaSorin TrimericS assay had the highest rate of positive results (66%.) The lowest percentage was observed for the Euroimmun assay (24%). The positivity rates for the other investigated methods were approximately 30%.

Starting at day 20′ and until the end of the study, almost 100% of the results were positive in all of the methods studied. On day 240′ one sample was negative in two (DiaSorin TrimericS and Euroimmun) assays and three samples were negative in the Euroimmun assay.

**Figure 4:**
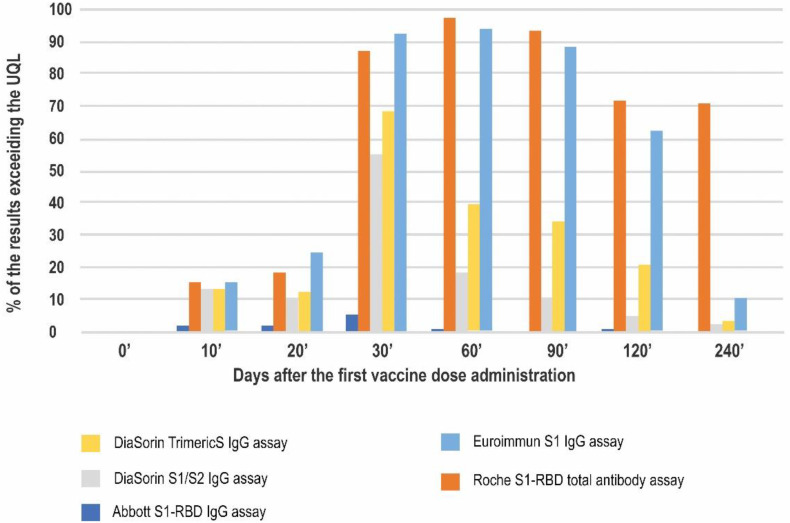
The percentage of the results exceeding the upper quantitation limit of a given immunoassay at 8 timepoints of the study.

This study was performed in a standard clinical laboratory setting and the dilutions were not planned. A high number of results exceeding the upper quantitation limit (UQL) was observed (Figure 4). The highest percentage of such results was seen in the Roche method, reaching 96.7% on day 60′ and sustaining at approximately 70% on days 120′ and 240′. Similarly, the Euroimmun assay reached its UQL on day 60′ in 94% of participants. However, this decreased to 10.6% on day 240′. 

At the peak of the vaccination-induced response (day 30′), more than half of the results in four out of the five methods studied exceeded the respective upper quantitation limits. The lowest number of such results was noted for the Abbott test (5.3%).

We also compared the percentages of the results reaching the arbitrarily chosen cut-off for a high result, which was proposed to be 10.9 times higher than the respective cut-off for a positive result for each method.

**Figure 5:**
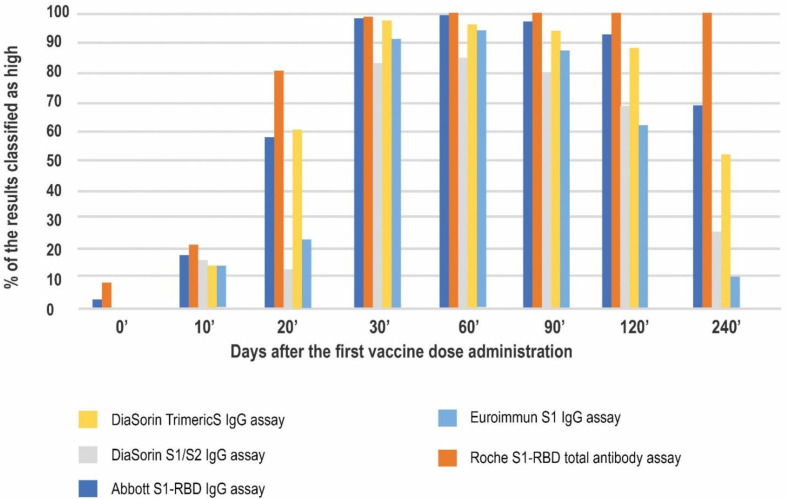
The percentage of results that were classified as high based on the arbitrary chosen cut-off.

The highest percentage of the high results was seen for the Roche test at all of the timepoints studied, reaching 100 percent on day 60′ and sustaining at this level until day 240′. On day 10′ all of the methods showed comparable ratios of high results (10–20%), mostly in the convalescent subjects. On day 20′, just before the second dose administration, the lowest percentage of the high results was noted for the DiaSorin S1/S2 assay (13%). In contrast, the other DiaSorin test reported up to 61% high results. The peak of the humoral response on days 30′, 60′, and 90′ (up to approximately two months after the second dose administration) was characterized by a substantial percentage (over 80%) of high results in all the methods. Over the course of the study the percentage of results classified as high dropped the most for the Euroimmun assay (to approximately 62% at four months and 11% at eight months after the first vaccination) (Figure 5). 

## 4. Discussion

There are a few dozen CE-IVD marked SARS-CoV-2 antibody immunoassays on the market. This burden may cause confusion for laboratory managers who need to decide which test to use in their everyday practice. At the beginning of the pandemic, the main aim of serological testing was to confirm COVID-19. There were discussions as to which tests perform better, nucleocapsid (N) or spike (S) protein-based [22,23]. The vaccine rollout at the end of 2020 worked in favor of the anti-S, quantitative assays, as the assessment of the humoral response to the COVID-19 vaccination and its possible role in the estimation of the protective immunity started to be crucial. The tests’ manufacturers considered the vaccines’ composition [24,25,26,27] and the protective role of anti-spike antibodies [28], and switched the specificity of the assays from detecting anti-N antibodies (observed only in COVID-19 convalescents) to detecting anti-S antibodies (observed in both convalescents and vaccinees). 

The aim of our study is to depict the performance of some of the anti-S SARS-CoV-2 immunoassays most commonly used in European medical laboratories in the context of the response to COVID-19 vaccination. The antibody composition in COVID-19 vaccinees may be different from that observed in convalescents [29]. To our knowledge, this is one of the very few papers encompassing head-to-head comparison of five methods over such a long course (eight months) after primary immunization. This study’s added value includes the multiple timepoints at which the humoral response to the vaccination was assessed.

All of the studied methods proved to detect the individuals’ responses to each of the two doses of COVID-19 vaccine, as well as the waning of humoral immunity eight months after the first vaccine dose injection (Figure 1, Table 2). The overall kinetics were in agreement with the typical course of the response following immunization and similar to that reported by Ferrari et al. in the context of COVID-19 vaccination over a six month course [30]. 

The results obtained using the investigated methods correlated strongly (Figure 2). The correlation between the immunoassays was previously reported by other researchers [31,32,33,34]; however, the strength of correlation varies between the papers, which may be attributed to the different timepoints investigated, the samples’ dilutions, and conversion to BAU/mL. Three of the methods included in our study were also compared by Lukaszuk et al. in a similar group of vaccinees. These authors reported overall correlations between the assays to be moderate to high: r = 0.663 for DiaSorin TrimericS–Abbott; r = 0.684 for DiaSorin TrimericS–Roche; and r = 0.902 for Roche–Abbott [31]. These values are lower than the Spearman’s coefficient of correlation observed in our study (r between 0.8 and 0.948). These differences may be the result of the study design. Our investigation covered a wider time frame, while Lukaszuk et al. analyzed samples obtained mainly at the peak of the humoral response to COVID-19 vaccination: prior to the first dose, on the day of the second dose, and 8, 14, and 30 days after the second dose (relative to days 0′, 20′, 30′, and 60′ of our study). Additionally, samples dilutions were performed.

In our study, the highest overall correlation was observed for the DiaSorin TrimericS–DiaSorin S1/S2 assays. The DiaSorin assays are the most similar in this study with respect to antigenic specificity and antibody class, and this is probably the reason for their highest overall correlation. However, in contrast to Infantino, for example, our results do not fully support the observation that the lowest agreement can be found for comparisons of kits using different antigenic targets [35]. For example, the overall correlation coefficients for the S1-RBD IgG Abbott assay and the TrimericS DiaSorin IgG assay are higher than for the S1-RBD Euroimmun IgG assay and the S1-RBD Roche total antibody assay (r = 0.919 vs. r = 0.832). The Roche test showed the lowest correlations with all of the methods, possibly due to the fact that only this assay detects total anti-SARS-CoV-2 S1-RBD antibodies, not just the IgG fraction. Perkmann et al., who assessed the correlations between the immunoassays 21 days after the first vaccine dose administration, had very similar observations regarding the Roche test’s lower agreement with other methods [36]. Similarly, Carta et al., who compared four SARS-CoV-2 anti-S immunoassays at three timepoints in 70 vaccinees, showed the lowest correlation between the results of the Roche and DiaSorin TrimericS assays [34]. Infantino et al., in their dataset from a mixed population (vaccinated subjects, patients recovered from COVID-19, and healthy individuals), also reported the lowest correlation coefficient value between the Roche S1-RBD total antibody and DiaSorin TrimericS IgG assays [35].

The lack of conversion to BAU/mL did not result in the lower correlation between the methods, as shown by the inclusion of the DiaSorin’s S1/S2 assay, already replaced on the market by the TrimericS assay.

The results of the other four methods included in this study were reported in Binding Antibody Units, which were developed to facilitate comparisons between the SARS-CoV-2 antibody assays [16]. Despite that, the numerical values of the median antibody concentrations at the given timepoints in our study were completely different between methods. For example, the median antibody concentrations observed on day 20′ (reflecting the response to only one dose of the vaccine) ranged from 31.74 BAU/mL (Roche assay) to 435.5 BAU/mL (DiaSorin TrimericS assay). This may be at least partially attributed to differences in the exact specificity of the studied immunoassays.

Another important issue we encountered was the high percentage of results exceeding the upper quantitation limit of each test. The sample dilutions eventually proposed by the manufacturers pose a few problems, including non-linearity of some samples’ dilutions, an increase in cost, and testing complexity for the laboratory. Therefore, we consider the immunoassays that produce a low percentage of results exceeding the UQL for undiluted samples to be more useful in the current setting.

The assay that proved to be the most resistant to the above issue in our study was the Abbott’s S1-RBD IgG test; approximately 1.6% of all samples displayed results higher than the UQL of 5680 BAU/mL. Such results were seen no later than 60 days after vaccination, and mostly in COVID-19 convalescents. The Abbott’s assay’s quantitation range is wide (2.98–5680 BAU/mL) and characterized by a high ratio of UQL/positive result cut-off (800).

In contrast, the highest number of results exceeding the UQL was found for the Roche test. The median values of antibody concentrations were higher than the assay’s UQL starting at day 30′ and until the end of this study. Therefore, the ability of this test, used without dilutions, to show the dynamics of antibody changes after vaccination, is compromised. A similar problem was observed for the Euroimmun’s S1 IgG assay, which showed a very high percentage of results above the UQL between days 30′ and 120′. However, between days 120′ and 240′ this percentage dropped from 71.0 to 10.6, and the results for 5% of the samples were already below the cut-off for a positive result. 

The question of the correlates of protection unsurprisingly captured attention. It is recognized that SARS-CoV-2 immunity is multi-dimensional and includes humoral and cellular response, as well as B- and T- memory cells [37]. Nonetheless, the serum antibody measurements remain the most accessible means of immunity assessment, and their presence in vaccine-naive healthcare workers was associated with a substantially reduced risk of SARS-CoV-2 reinfection in the ensuing six months [14].

As mentioned by Tang et al., some data point to serum neutralizing titers >1:250 being protective against COVID-19 [38]. It was established that anti-spike antibodies carry this neutralizing function crucial for immunity [28]; higher antibody levels were reported to correlate with reduced risk of a symptomatic infection [39].

The unified thresholds for antibody concentrations that can be classified as high, and therefore expected to have pronounced neutralizing activity, are not yet defined [40,41,42]. For the purpose of this study we developed a surrogate definition of a high titer–constant multiple of an immunoassay-respective cut-off for seropositivity that is quantifiable in all of the methods investigated. 

Based on data from the tests’ manufacturers and research papers [39,43], high neutralization capabilities are observed for antibody concentrations 5–600 times higher than the positivity cut-off values. This encompasses our 10.9 multiplier, computed as the lowest ratio of UQL/positivity cut-off. This 10.9 multiplier was based on the Euroimmun’s immunoassay; therefore, only the results exceeding the UQL were considered high for this assay. Since there are no Euroimmun-provided data on the concentrations correlated to the neutralization titers, no conclusions may be drawn regarding this assay’s capability to indicate protection.

The adjusted, calculated cut-off for the Roche’s immunoassay, 9 BAU/mL, is lower than the manufacturer-provided concentration correlated to high neutralizing capability, 15.43 BAU/mL (18× higher than the positivity threshold). However, it is unlikely that either of these values are indicative of protection. In this study, 100% of the Roche’s results between days 60′ and 240′ were classified as high or with high neutralizing capability. Yet, the clinical observations indicate that the probability of break-through infections is a factor of time from vaccination [44]; therefore, it may be expected that 240 days after immunization (approximately seven months after the second dose) some of the vaccinees will suffer from COVID-19. This is mirrored by the percentages of high results obtained using the other methods dropping significantly by day 240′ of our study. Further, one of the first papers correlating the antibody titers with symptomatic COVID-19 reported that for S1-RBD assays the concentration that might be a surrogate marker of protection is approximately 500 BAU/mL (approximately 600× higher than the Roche’s positivity cut-off) [39]. This value is much higher than the Roche’s assay quantitation range. For this reason, testing undiluted samples using this method is not suited to deciphering immunity status, especially considering that the percentage of samples exceeding the UQL on day 240′ is still as high as 70. 

When the DiaSorin’s methods are compared, the TrimericS IgG immunoassay reports higher percentages of the high results than the S1/S2 assay. The manufacturer showed that its TrimericS assay result of 520 BAU/mL (15.4× the positivity cut-off) correlated with the microneutralization titer of 1:80. This is slightly higher than our calculated threshold for the high result (368 BAU/mL). However, both values are much lower than the 1300 BAU/mL suggested for this technology by Padoan et al. [43] as highly protective (correlating to the PRNT titer 1:320). The DiaSorin’s S1/S2 IgG assay result of 80 AU/mL (only 5× the positivity cut-off; hence, lower than the 163 AU/mL considered high in our study), according to its manufacturer, indicates a titer of 1:160 in a plaque reduction neutralization test (PRNT). Nevertheless, both DiaSorin methods’ estimations of between 25–50 percent of the high results on day 240′ seem to be reasonable, and are between the Roche (all high results) and the Euroimmun (10% of such results) immunoassays. 

The concentration classified as high in our study for the Abbott’s technology was relatively low, 77 BAU/mL, with a 65% rate of high antibodies on day 240′. The concentration shown by its manufacturer to correspond to the PRNT dilution of 1:80 was 136 BAU/mL (19× higher than the cut-off for the positive result) and to the PRNT titer of 1:160 was 490 BAU/mL (69× higher). 

It must also be noted that the concentrations considered high in our study were much lower than 899 BAU/mL for spike-based assays (in our study, the Euroimmun’s and both DiaSorin’s assays) and 2360 BAU/mL for RBD-based assays (the Roche’s and Abbott’s assays), which correlated to 90% protection against a symptomatic SARS-CoV-2 infection over the following four to six months, as noted by Feng et al. [39].

The above data indicate that the cut-off multiplication by a constant factor, aimed at classifying the concentration of antibodies as high or neutralizing, is not fully adequate for all of the methods investigated. The differences between the adjusted, manufacturer-provided cut-offs and the research data highlight the need for standardization of future studies regarding antibody concentrations potentially indicating neutralizing capacity, and ultimately, SARS-CoV-2 protection. Currently, it may only be stated that medium high titers may be enough to protect against severe infection, whereas very high titers may be enough to protect against any infection [45].

## 5. Conclusions

To sum up the practical implications of our study, it may be concluded that the Abbott’s assay, exhibiting the widest measurement range, the lowest percentage of results exceeding the UQL and sufficient agreement between manufacturer-provided concentration correlated to the neutralizing ability and the published data, seems to be well suited for anti-SARS-CoV-2 antibodies measurement in the context of COVID-19 vaccination. In contrast, the Roche method shows the weakest correlations with the other methods; most of the results of this assay exceeded the UQL and the concentration of the antibodies tentatively suggestive of neutralization capabilities are probably even higher than the UQL (in strong disagreement with the manufacturer-provided data). The DiaSorin’s TrimericS assay, which replaced the Diasorin S1/S2 test, correlates very well with the Abbott’s immunoassay; however, it produces a higher percentage of results exceeding the UQL. The Euroimmun’s test, as typical ELISA methodology, is not feasible in the routine laboratory its measurement range is narrow, and in our study many results exceeded the UQL between 30 and 120 days after the vaccination.

Our study presents the results of quantitative anti-SARS-CoV-2 spike antibodies testing in COVID-19 vaccinees at eight timepoints over eight months following administration of the first dose. Although the five investigated methods correlate strongly with each other, single measurements are different between the assays, even after conversion to standardized units (BAU/mL). Further, our study provides evidence of extremely high antibody concentrations after vaccination, very often exceeding the linearity range of commonly used immunoassays. The results of our study were not meant to reveal the holy grail of the serology testing in the context of COVID-19—the protective antibody titer. Yet, we showed that attempts to interpret immunity status based on measured antibody concentration and its correlation to the neutralization titer may be in vain, as it leads to contrary conclusions depending on the methodology and the cut-offs used.

## Figures and Tables

**Table 1:** The characteristics of 5 immunoassays studied.

	Antibody Class	Antigen	Conversion to BAU/mL	LQL [BAU/mL]	Positive Result Cut-off [BAU/mL]	UQL	UQL/Positive Cut-off	High Result Adjusted Cut-off (10.9× Positive Cut-off)
DiaSorin TrimericS IgG assay	IgG	Trimeric S	AU/mL × 2.6	4.81	33.8	2080	61.54	368
Abbott S1-RBD IgG assay	IgG	S1-RBD	AU/mL × 0.142	2.98	7.1	5680	800	77
Roche S1-RBD total antibody assay	Total Ab	S1-RBD	U/mL: 0.972	0.41	0.82	257.2	313.6	9
Euroimmun S1 IgG assay	IgG	S1	RU/mL × 3.2	3.2	35.2	384	10.9	384
DiaSorin S1/S2 IgG assay	IgG	S1/S2	n/a	3.8 AU/mL	15 AU/mL	400 AU/mL	26.67	163

LQL—Lower Quantitation Limit; UQL—Upper Quantitation Limit. DiaSorin S1/S2 IgG assay has not been validated against WHO-IS and this assay’s characteristics are given in AU/mL.

**Table 2:** Anti-SARS-CoV-2 antibody serum concentrations in 100 vaccinees measured by 5 immunoassays at 8 timepoints over 8 months following the first vaccine dose administration.

	Median Antibody Concentration [BAU/mL] (Min–Max)							
	**0′**	**10′**	**20′**	**30′**	**60′**	**90′**	**120′**	**240′**
DiaSorin TrimericS IgG assay	4.81 (4.81–256)	93.21 (4.81–2080)	435.5 (18.4–2080)	2080 (100.62–2080)	1931.8 (186.94–2080)	1669.2 (116.47–2080)	1090 (46–2080)	379 (26.4–2080)
Euroimmun S1 IgG assay	3.2 (3.2–132)	6.6 (3.2–384)	202.8 (5.9–384)	384 (133.3–384)	384 (116.4–384)	384 (149.2–384	384 (29.2–384)	178.05 (19.2–384)
DiaSorin S1/S2 IgG assay (results expressed in AU/mL)	3.8 (3.8–72.7)	7.0 (3.8–400)	74.05 (3.8–400)	400.0 (17.2–400)	247 (60–400)	223 (67.4–400)	194 (30.2–400)	117 (18.2–400)
Roche S1-RBD total antibody assay	0.4 (0.41–161.5)	0.4 (0.41–257.2)	31.74 (0.41–257.2)	257.2 (2.12–257.2)	257.2 (13.7–257.2)	257.2 (75–257.2)	257.2 (18.11–257.2)	257.2 (35.19–257.2)
Abbott S1-RBD IgG assay	2.98 (2.98–93.79)	4.07 (2.98–5680)	83.95 (2.98–5680)	1471.8 (11.94–5680)	1075.99 (16.06–5680)	490.26 (56.25–3034.85)	236.71 (9.81–5680)	110.115 (12.68–2898.75)

## Data Availability

The data presented in this study are available on reasonable request from the corresponding author.
